# Effectiveness of auricular laser acupuncture on anxiety, stress, sleep quality, fatigue and muscle tension of Psychosocial Care Center professionals: a quasi-experimental pilot study

**DOI:** 10.1590/0034-7167-2024-0534

**Published:** 2025-07-11

**Authors:** Lara Rocha Silva, Luana Vieira Toledo, Andreia Guerra Siman, Talita Prado Simão Miranda, Maria Clara Vidgal Santana, Yara Martins Rodrigues, Bruna de Oliveira Alves, Caroline de Castro Moura

**Affiliations:** I Universidade Federal de Viçosa. Viçosa, Minas Gerais, Brazil; II Universidade Federal de Minas Gerais. Belo Horizonte, Minas Gerais, Brazil

**Keywords:** Acupuncture, Ear, Light Therapies, Low-Level, Mental Health Services, Anxiety, Stress, Psychological., Acupuntura Auricular, Terapia por Luz de Baja Intensidad, Servicios de Salud Mental, Ansiedad, Estrés Psicológico.

## Abstract

**Objectives:**

to assess the effectiveness of auricular laser acupuncture in professionals from Psychosocial Care Centers regarding anxiety, stress, sleep quality, fatigue, trapezius muscle tension threshold, satisfaction with treatment and possible adverse reactions.

**Methods:**

a quasi-experimental pilot study conducted with 24 professionals. Five auricular laser acupuncture sessions were performed. Data were collected at the beginning and end of treatment, and in a period of 15 days after the end (follow-up), analyzed by paired Wilcoxon test.

**Results:**

auricular laser acupuncture was effective in reducing levels of anxiety, stress, fatigue and improving sleep latency. There was no statistically significant difference in muscle tension threshold. Professionals were satisfied with treatment, and the occurrence of adverse reactions was not significant.

**Conclusions:**

auricular laser acupuncture triggered positive effects for all variables investigated, except for trapezius muscle tension threshold.

## RESUMEN

**Objetivos:**

evaluar la efectividad de la auriculoacupuntura láser en profesionales de Centros de Atención Psicosocial respecto a ansiedad, estrés, calidad del sueño, fatiga, umbral de tensión del músculo trapecio, satisfacción con el tratamiento y posibles reacciones adversas.

**Métodos:**

estudio piloto cuasiexperimental, realizado con 24 profesionales. Se realizaron cinco sesiones de ariculoacupuntura láser. Los datos fueron recolectados al inicio y al final del tratamiento, y en un período de 15 días posteriores a su finalización (*follow-up*), analizados mediante la prueba de Wilcoxon pareada.

**Resultados:**

la auriculoacupuntura láser fue efectiva para reducir los niveles de ansiedad, estrés, fatiga y mejorar la latencia del sueño. No hubo diferencias estadísticamente significativas en el umbral de tensión muscular. Los profesionales quedaron satisfechos con el tratamiento y la aparición de reacciones adversas no fue significativa.

**Conclusiones:**

la auriculoacupuntura láser desencadenó efectos positivos para todas las variables investigadas, excepto el umbral de tensión del músculo trapecio.

## INTRODUCTION

The COVID-19 pandemic has caused significant changes in society, including for healthcare professionals, who, due to work overload and physical, intellectual and emotional demands, have had a negative impact on their quality of life. In this context, Psychosocial Care Center (In Portuguese, *Centro de Atenção Psicossocial* - CAPS) professionals stand out, who, even before the pandemic, already had high levels of emotional disorders, such as anxiety^(1)^ and stress^(2)^.

Anxiety and stress affect the body’s homeostasis and cause signs and symptoms that are perceived differently by individuals. These include changes in sleep quality, fatigue and muscle tension^(3)^_._

Scientific evidence points to a high prevalence of anxiety and stress^(4)^ in healthcare professionals during the COVID-19 pandemic. However, studies investigating the impact of these disorders on CAPS professionals during the pandemic and post-pandemic periods are scarce. In this context, it is essential to understand the impact of COVID-19 on the mental health of these professionals and how this has affected the post-pandemic period, in order to implement measures to protect and improve the quality of their lives^(5,6)^. Integrative and Complementary Health Practices (ICHPs), which are institutionalized in the Brazilian Health System^(7)^, especially auriculoacupuncture, have stood out in this scenario.

Auriculoacupuncture is a therapeutic acupuncture technique that involves stimulating energy points in the ear, where the entire organism is represented, through a microsystem^(8)^. When an ear point is stimulated, peptides and neurotransmitters are released centrally, triggering the therapeutic action of the intervention at a systemic level^(9)^. This technique contributes to improving anxiety, stress, depression^(10)^ and sleep quality^(11)^. Consequently, it also helps promote mental health.

Several devices can be used to stimulate auricular acupoints, such as crystals, needles, seeds and low-intensity laser. The laser is a non-invasive, aseptic and comfortable device for users^(12)^. However, scientific literature is scarce on the use and effectiveness of auricular laser acupuncture in emotional disorders in CAPS professionals, indicating a little explored scientific reality.

## OBJECTIVES

To assess the effectiveness of auricular laser acupuncture in CAPS professionals regarding anxiety, stress, sleep quality, fatigue, trapezius muscle tension threshold, satisfaction with treatment and possible adverse reactions.

## METHODS

### Ethical aspects

The study was conducted in accordance with national^(13)^ and international^(14)^ ethics guidelines and was approved by the *Universidade Federal de Viçosa* Research Ethics Committee, whose opinion is attached to this submission. The Informed Consent Form was obtained from all individuals involved in the study in writing in two copies, one remaining with the researcher and the other with participants.

### Study design, place and period

This is a quasi-experimental pilot study, with assessment of a single group before and after intervention, reposted according to the Transparent Reporting of Evaluations with Nonrandomized Designs (TREND) statement^(15)^, and carried out with CAPS I, II and AD III professionals in two cities in the *Zona da Mata Mineira* between August 2023 and July 2024.

### Population and sample; inclusion and exclusion criteria

The study population consisted of all 53 professionals who worked at CAPS. The sample was obtained by convenience, as all professionals were invited to collaborate with the research.

Professionals with time availability to participate in intervention and data collection were included. Professionals who had ear piercings, except in the earlobe, history of photosensitivity, injury, inflammation, tattoo or deformity in the ear, use of hearing aids, compromised immune system, epilepsy, use of cardiac pacemaker, pregnant women, postpartum women or women who planned pregnancy during the study period, performance of another ICHP concomitantly with intervention and leave or vacation during data collection were excluded. Professionals who developed an allergic or inflammatory reaction in the ear, missed two consecutive sessions or exceeded the interval greater than ten days between them or who did not correctly complete the data collection instruments were discontinued from the study.

### Study protocol

Participants were recruited via virtual invitation and during team meetings. Those who met the eligibility criteria and agreed to participate in the research signed the Informed Consent Form.

Auriculoacupuncture was performed using a low-intensity diode laser with a wavelength of 808 nanometers (infrared), average power of 100 mW and a beam spot area of 1 cm^2^ (Therapy ACP - DCM^®^). The laser was applied in contact with the skin, continuously, punctually (perpendicular to the skin) and stationary. Application time was 40 seconds for each stimulated point, with a total intervention time of six minutes, with an energy dose of 4 joules (irradiance per point: 100 mW/cm^2^; fluence per point: 4.0 J/cm^2^)^(16)^. A spacer (area: 0.09842 cm^2^; diameter: 3.54 mm - DMC^®^) was used at the tip of the laser cannula in order to maintain a safe distance between the cannula and the participant’s skin. Before laser application, a polyvinyl chloride film was placed on the spacer and cannula of the equipment for greater hygiene, which was changed after each session. During the entire laser application, the participant and the interventionist wore protective glasses made of polycarbonate with a special dielectric film arrangement to attenuate the lasers.

Five sessions of auricular laser acupuncture were performed, one per week, with alternating ears in each session. A participant was positioned comfortably seated in a chair, and the ear was inspected to detect any changes that would prevent the technique application. The area was then antisepsised with 70% alcohol and cotton. A total of nine acupoints, scientifically validated in healthcare professionals with emotional disorders in the pandemic context^(17)^, were irradiated in all participants, in all sessions: *Shenmen*; Sympathetic Nervous System; Kidney; Liver; Spleen; Brainstem; Upper Lung; Heart; and Liver Yang 2. The auricular map recommended by the World Federation of Acupuncture-Moxibustion Societies^(18)^ was used for the correct location of the acupoints. The intervention was performed by a nurse specializing in Chinese auriculoacupuncture, with six months of training in the area, who was duly trained for the correct location and irradiation of the acupoints by a nurse specializing in acupuncture with 12 years of experience in the area.

Anxiety and stress were the primary outcomes of this study, and sleep, fatigue, and trapezius muscle tension threshold were considered secondary outcomes

Anxiety was assessed using the State-Trait Anxiety Inventory, which has been validated for Brazil^(19)^ and assesses the momentary emotional state. It has 20 items with a four-point Likert scale, ranging from one (absolutely not) to four (very much)^(19)^. The score ranges from 20 to 80 points, with low anxiety levels (20-30), medium anxiety levels (31-49) and high anxiety levels (≥50)^(20)^.

Stress was measured using the Perceived Stress Scale^(21)^, which was translated and validated for Brazil^(22)^. This scale assesses 14 items, ranging from one (almost never) to four (very often). The total score is given by the sum of the questions, and can range from zero to 56, with stress levels being low (≤18), normal (19-24), moderate (25-29), high (30-35) and very high (>35)^(22)^.

Sleep quality was assessed using the Pittsburgh Sleep Quality Index, which was translated and validated for Brazil^(23)^. The questionnaire has 19 self-assessment questions, five of which are to be answered by a bed partner or roommate. The 19 questions are categorized into sleep components, such as subjective quality, sleep latency, sleep duration, sleep disorders, medication use, and daily dysfunction, and each is graded on a scale of zero to three. The sum of the overall score ranges from zero to 21, and scores above five indicate poor sleep quality^(23)^. For the present research, the 19 self-assessed questions were used.

Fatigue was assessed using the Fatigue Assessment Scale, which was translated into Brazilian Portuguese^(24)^ and validated for the context of healthcare professionals^(25)^. The scale consists of ten items with scores ranging from one (never) to five (always). The final score is given by adding the items together, and can range from ten to 50. The higher the score, the greater the signs of fatigue^(24)^.

Trapezius muscle tension threshold was measured using a digital algometer (DDK, Kratos^®^)^(26)^. For this assessment, a participant sat in a chair with the chest region supported by the backrest, arms flexed on the backrest, head erect, legs uncrossed and feet completely supported on the floor. Pressure was applied to four points in the trapezius region, perpendicular to the skin, in the following locations: midpoint of the descending trapezius (between the acromion and the 7^th^ cervical vertebra) bilaterally; and a point between the 5^th^ and 6^th^ cervical vertebrae, in the descending trapezius muscle, bilaterally. A participant was asked to press the interrupting cable of the device when they felt that the mechanical stimulus became painful. Each point was assessed in triplicate, and the mean of the points was used for analysis.

The following covariates were also considered in this study: professional characterization variables, adapted from Assis *et al*.^(17)^; the influence of the COVID-19 pandemic on mental health and professional performance (no influence, influence for the better or influence for the worse); the feeling of work overload after the pandemic; satisfaction with treatment, through three questions (satisfaction with intervention, need for intervention and change in general health status), on a five-point Likert scale; and possible adverse reactions resulting from intervention application (hyperemia, edema or scaling in the ear and headache), which were evaluated on a scale from zero (no discomfort) to ten (severe discomfort).

Data collection occurred at three moments: before starting intervention (initial assessment); one week after the last intervention session (final assessment); and 15 days after the last intervention (follow-up assessment). The instruments were self-completed, and the muscle tension assessments were performed in person by the same researcher, who was properly trained.

### Analysis of results, and statistics

The collected data were analyzed using the Statistical Package for the Social Sciences version 23. Descriptive statistics were used to describe and summarize the variables studied. To verify data distribution, the Shapiro-Wilk test was used, which showed the non-normal distribution of scalar variables. The primary and secondary outcome variables, at different time intervals (initial, final and follow-up assessments), were analyzed using the paired Wilcoxon test at 5% significance. To calculate the effect size, Cohen’s D test was used using GPower 3.1.9.2, which can be classified as: d≤0.2 - small or null effect size; 0.3<d<0.8 - medium effect size; d≥0.8 - large effect size^(27)^.

## RESULTS

Of the 32 professionals who met the eligibility criteria, 24 (75.0%) completed the intervention protocol until the final stage (25.0% loss), and 20 (62.5%) until the follow-up stage (37.5% loss).

Of the 24 professionals who completed the intervention, the majority were women (66.7%), white (45.8%), single (45.8%), Catholic (70.8%) and had no children (54.2%). The median age was 36 years (20-62), and the median length of service was 13.5 months (6.2-52.5). In addition, 45.8% worked at CAPS I, 29.2% at CAPS II, and 25.0% at CAPS AD III. Of these, 54.2% had two or more jobs, and 33.3% were permanent employees. Additionally, 91.7% worked during the COVID-19 pandemic, and 54.2% contracted the disease. Concerning clinical conditions, 16.7% had some anxiety disorder; 8.3% used anxiolytics; 20.8% used antidepressants; 66.7% consumed alcohol; and 29.2% were smokers. In addition, 58.3% of professionals reported that the pandemic negatively influenced their mental health; 29.2% reported that the pandemic negatively influenced their professional performance; and 41.7% felt more overwhelmed at work after the pandemic.

There was a statistically significant reduction in the level of state anxiety between the initial and follow-up assessments, with a medium effect size. There was also a statistically significant reduction in the level of stress between the initial and final assessments and between the initial and follow-up assessments, with a medium effect size in both assessments. In sleep quality assessment, a statistically significant improvement in the sleep latency component was also evidenced between the initial and final assessments, with a small effect size. Furthermore, there was a statistically significant improvement in fatigue between the beginning and end of treatment, with a medium effect size. There were no statistically significant changes in trapezius muscle tension threshold throughout treatment (Table 1).

**Table 1:** Levels of state anxiety, stress, fatigue, sleep and muscle tension at baseline, final and follow-up assessments (n=24), Viçosa, Minas Gerais, Brazil, 2024

	IA n=24		FA n=24		FWA n=20		*p* value^||^ (effect size)		
**Variables**	**Mean** **(SD)**	**Median** **(IR)**	**Mean** **(SD)**	**Median** **(IR)**	**Mean** **(SD)**	**Median** **(IR)**	**FA** **IA**	**FWA** **FA**	**FWA** **II**
**State anxiety**	45.13 (9.65)	45.00 (38.75-50.50)	42.42 (9.93)	41.00 (36.00-51.00)	39.65 (9.36)	39.50 (33.25-46.75)	0.053 (0.272)	0.730 (0.286)	0.017 (0.566)
**Stress**	30.71 (9.10)	30.50 (23.75-38.00)	25.67 (7.90)	27.00 (19.00-33.00)	24.35 (6.74)	25.00 (18.25-30.75)	0.001 (0.588)	0.635 (0.178)	0.005 (0.777)
**Sleep quality**									
General sleep quality	8.54 (3.98)	9.00 (6.00-10.00)	7.36 (3.88)	7.00 (4.50-9.75)	7.45 (3.33)	8.00 (5.25-9.00)	0.560 (0.300)	0.750 (0.024)	0.216 (0.294)
Subjective sleep quality	1.46 (0.78)	1.00 (1.00-2.00)	1.33 (0.64)	1.00 (0.00-3.00)	1.20 (0.52)	1.00 (1.00-1.75)	0.405 (0.180)	0.414 (0.339)	0.317 (0.377)
Sleep latency	1.71 (1.20)	2.00 (0.25-3.00)	1.38 (1.01)	1.50 (0.25-2.00)	1.40 (0.99)	1.00 (1.00-2.00)	0.035 (0.295)	0.655 (0.019)	0.096 (0.279)
Sleep duration	1.04 (0.86)	1.00 (0.00-2.00)	0.88 (0.74)	1.00 (0.00-1.00)	0.95 (0.83)	1.00 (0.00-1.00)	0.285 (0.198)	0.180 (0.088)	0.729 (0.106)
Sleep efficiency	0.71 (0.75)	1.00 (0.00-1.00)	0.79 (0.93)	0.50 (0.00-1.75)	0.95 (1.05)	0.50 (0.00-2.00)	0.644 (0.093)	0.257 (0.160)	0.417 (0.256)
Sleep disturbance	1.79 (0.93)	2.00 (1.00-2.00)	1.54 (0.78)	1.00 (1.00-2.00)	1.55 (0.60)	1.50 (1.00-2.00)	0.083 0.289	0.414 (0.014)	0.134 (0.293)
Medication use	0.63 (1.13)	0.00 (0.00-1.00)	0.54 (0.98)	0.00 (0.00-1.00)	0.40 (0.94)	0.00 (0.00-0.00)	0.726 (0.084)	0.414 (0.145)	0.581 (0.219)
Sleepiness and daytime dysfunction	1.21 (0.93)	1.00 (0.25-2.00)	1.00 (0.78)	1.00 (0.25-1.00)	1.00 (0.72)	1.00 (0.25-1.75)	0.212 (0.242)	0.705 (0.001)	0.405 (0.248)
**Fatigue**	24.83 (5.35)	24.00 (20.25-28.75)	22.79 (4.36)	22.50 (20.00-25.00)	22.50 (3.65)	22.00 (21.00-24.00)	0.020 (0.413)	0.625 (0.123)	0.190 (0.492)
**Trapezius muscle tension**	4.17 (1.40)	4.14 (3.18-5.30)	3.80 (1.38)	3.84 (2.97-5.08)	3.82 (1.49)	3.86 (2.60-5.40)	0.103 (0.266)	0.884 (0.01)	0.108 (0.241)

*IA - initial assessment; FA - final assessment; FWA - follow-up assessment; n - absolute frequency; ||paired Wilcoxon test; SD - standard deviation; IR - interquartile range.*

Most professionals (79.1%) were satisfied or extremely satisfied with intervention, and considered treatment necessary or totally necessary (79.1%). Furthermore, after completion, 62.5% considered that their general health status was better (Table 2).

**Table 2:** Satisfaction, need for intervention and general health status of study participants (n=24), Viçosa, Minas Gerais, Brazil, 2024

Variables	n	%
Satisfaction with intervention		
Extremely dissatisfied	0	0.0
Dissatisfied	0	0.0
Unsure	5	20.8
Satisfied	14	58.4
Extremely satisfied	5	20.8
Need for intervention		
Totally unnecessary	0	0.0
Unnecessary	0	0.0
Not sure	5	20.8
Necessary	14	58.4
Totally necessary	5	20.8
General health status after intervention		
Much worse	0	0.0
Worse	0	0.0
No change	9	37.5
Better	15	62.5
Much better	0	0.0

*n - absolute frequency; % - relative frequency.*

Finally, only one professional (4.2%) reported headache (intensity=2/10) after the second session of auricular laser acupuncture. The others (95.8%) did not present adverse reactions.

## DISCUSSION

It was demonstrated, with the performance of this study, that auricular laser acupuncture was able to reduce the levels of state anxiety, stress and fatigue, in addition to providing an improvement in sleep quality, specifically in the sleep latency component, in CAPS professionals, in the post-COVID-19 pandemic, during the established treatment period. Most professionals were satisfied with intervention, considering it necessary and reporting an improvement in their general health after treatment. Furthermore, the occurrence of adverse reactions was not significant.

The findings of this study are relevant because they involved a population that is little investigated in scientific literature, especially in relation to the occurrence of emotional disorders. Furthermore, the use of laser is an innovation in treatment through auriculoacupuncture, and its action on emotional factors is little explored. In fact, a systematic review^(10)^ aimed at identifying the effects of auricular acupuncture in the treatment of stress, anxiety and depression revealed that, of the 24 articles presented, only one addressed auricular laser acupuncture for physical and emotional symptoms of temporomandibular dysfunction^(16)^, showing that this is an area that still requires research.

Neurophysiology explains the action of auriculoacupuncture through modulation of the hypothalamic-pituitary axis^(28)^. It is believed that stimulation of acupoints triggers biochemical signals that promote changes in brain structures, such as the limbic system and the prefrontal cortex^(29)^, contributing to the reduction of anxiety and stress, for instance.

Worsening anxiety was one of the deleterious effects on the mental health of healthcare professionals during the COVID-19 pandemic^(30)^, and its effects are still noticeable. After auricular laser acupuncture, there was a statistically significant reduction of 12.1% in anxiety levels between baseline and follow-up. The findings corroborate a randomized clinical study that used the same protocol of auricular points and number of sessions, developed with 284 nursing professionals during the pandemic, which showed a 60% reduction in anxiety between the initial and final moments and 35.9% between the initial and follow-up moments, using needles^(17)^. In this regard, the effect triggered by needles, which are considered the gold standard devices in acupuncture, appears to be superior in reducing anxiety; however, studies comparing these interventions in the same population are needed to establish this relationship.

Stress showed a statistically significant reduction between the initial and final moments and the initial and follow-up moments, of 16.4% and 20.7%, respectively, going from high to moderate levels after treatment. A study with nursing professionals during the pandemic revealed a 38.3% reduction in this variable in a single session of auriculoacupuncture with seeds, using the *Shenmen*, Kidney, Sympathetic Nervous System, Joy, Anxiety, Antidepressant, Heart, Endocrine, Lung and Muscle Relaxation points^(31)^. A mixed methodology study, with a randomized quantitative and exploratory qualitative approach with the perioperative nursing team, showed a reduction in stress of 34.0% after treatment and 19.9% 15 days after intervention, compared to the initial moment^(32)^. This study used semi-permanent needles in *Shenmen,* Brainstem, Kidney, Sympathetic and Liver, in eight sessions, twice a week^(32)^. It is noteworthy that the *Shenmen*, Kidney and Sympathetic Nervous System acupoints were common in these studies. *Shenmen* has a sedative function; Kidney has an energetic and invigorating function; and Sympathetic Nervous System is responsible for regulating the sympathetic and parasympathetic nervous systems, with an effect on muscle relaxation and pain^(10)^. Thus, the combination of the three points, using seeds, needles, or laser, presents positive results for stress.

In relation to sleep quality, a statistically significant reduction of 18.8% was observed in the sleep latency component between the initial and final moments. It is worth noting that the two acupoints used in the present study, *Shenmen* and Heart, contribute to improving sleep, since they stimulate the vagus nerve and promote parasympathetic action in the body^(33)^, contributing to the relaxation necessary for sleep. In fact, a quasi-experimental study with hospital nurses during the pandemic demonstrated a 52.3% reduction in general sleep quality and 37.5% in average sleep latency, using neutral spheres with three sessions at the *Shenmen*, Kidney, Sympathetic Nervous System, Heart, Lung, Subcortex, Endocrine, Zero Point or Muscle Relaxation, and Adrenal points^(11)^. In China, a randomized double-blind study with 147 older adults found that magneto auricular acupuncture, auricular laser acupuncture and the combination of both therapies, on the *Shenmen*, Heart, Liver, Spleen, Kidney, Occipital and Subcortex acupoints, three times a week, for six weeks, significantly reduced the Pittsburgh Sleep Quality Index, being significant between the initial moment and the sixth month of follow-up in the population studied^(34)^. In this study^(34)^, a low-density laser was used continuously, with a wavelength of 650 nm, average output power of 2.5 mW, energy density of 0.54 J/cm^2^ for one minute, and each point was irradiated for approximately 60 seconds. Although the study presented different laser parameters from the present study, it is worth noting that both had positive effects on sleep quality. Furthermore, it seems that therapy with more sessions and shorter intervals between them is more beneficial for improving this variable and for its effects to last. However, to confirm this, it is suggested that clinical studies be carried out that compare different laser dosimetric parameters with the same auricular protocol.

Poor sleep quality can trigger fatigue^(35)^, and this can affect professionals’ psychological well-being, as happened during the COVID-19 pandemic^(36)^. In fact, a descriptive and cross-sectional multicenter study, developed with 2,667 nurses during the pandemic, identified a direct relationship between the degree of anxiety, perceived stress, depression and fatigue^(37)^. This research demonstrated a statistically significant reduction of 8.2% in fatigue at the end of treatment. In this context, these findings can be explained by the improvement in stress and anxiety levels through auricular laser acupuncture, which may have contributed to improving professionals’ disposition. It is worth noting that, to date, no study using this technique on fatigue levels in healthcare professionals has been identified in scientific literature, highlighting the innovative nature of this study. It is important to emphasize the importance of adopting measures to control fatigue in the workplace and, consequently, occupational diseases.

Increased muscle tension is one of the bodily manifestations caused by emotional disorders. Auriculoacupuncture showed significant improvement in algometer patterns in back pain with a protocol of five sessions, once a week, using needles^(38)^. The acupoints used were *Shenmen*, Kidney, Sympathetic Nervous System, Subcortex, Bladder, Liver and those corresponding to the pain site^(38)^. It is worth noting that, in this investigation^(38)^, the selected acupoints have analgesic and muscle relaxation action, which may have resulted in a better therapeutic effect on the pain threshold. In this sense, it is suggested that future research include acupoints with these functions, in order to verify the action of auricular laser acupuncture on the muscle tension threshold.

It is also worth noting that most professionals reported being satisfied with the intervention performed, which is a necessary therapy and produces improvements in general health status. In line with this, a study carried out with students during the pandemic also demonstrated great acceptability of the technique^(39)^, allowing its implementation in this population. A qualitative study with nursing professionals demonstrated that the fact that the procedure took place in work environments facilitated intervention implementation and that they felt recognized and valued^(40)^. This fact, combined with the use of laser, which allows the therapy to be painless, non-invasive^(41)^ and comfortable^(12)^, may have contributed to these findings. The high level of satisfaction with the treatment performed, associated with the effects triggered by intervention, may be important indicators to favor its implementation in the workplace as part of public policies in favor of worker health.

Furthermore, no participant had a serious adverse event. Only one participant had a headache, which was transient, tolerable and of low intensity, allowing treatment to continue. A randomized study, with the aim of verifying whether magneto and auricular laser acupuncture were effective in improving sleep, identified ear itching and acupoint sensitivity as adverse events of laser acupuncture, which were resolved spontaneously^(34)^. Thus, the use of laser in auriculoacupuncture has been consolidated as a safe practice, being associated with few adverse events, mild and transient^(42)^. Furthermore, its effectiveness is unquestionable^(12)^.

The findings of this research reinforce that auriculoacupuncture is beneficial in reducing anxiety and stress in healthcare professionals. The positive points include the precise dosage of the laser, the exposure of a small area, the ease of use, the minimum time required to perform the procedure and the ease of use^(12)^. Furthermore, it is aseptic, comfortable, non-invasive^(12)^ and painless^(42)^. This combination of benefits makes auricular laser acupuncture a promising therapy for healthcare professionals’ mental healthcare, with the possibility of being implemented in worker health promotion programs.

### Study limitations

Study limitations include sample size and convenience recruitment, which may interfere with the generalization of the findings. However, this was a pilot study conducted with a population that has not been investigated much, which may provide support for future clinical trials with larger samples. In addition to this, the high dropout rate of professionals, mainly due to dismissals and medical certificates, restricted the monitoring of a larger number of participants. Another limitation refers to the overload of professionals, resulting from the incomplete composition of the teams, which prevented interviews from being conducted during data collection. To overcome this situation, self-completion was adopted, however some sample losses occurred at this time.

### Contribution to health, nursing or public policy

The study findings provide support for nurses to incorporate auricular laser acupuncture into their care context. The appropriation of this ancient practice allows for a holistic view of the person. In addition, the use of laser offers a safe, painless and highly accepted therapy, enabling mass treatment, as it requires few materials for its implementation. In the context of CAPS work, this approach improves emotional disorders, contributing to professionals’ emotional well-being when dealing with situations that may compromise their mental health.

## CONCLUSIONS

Auricular laser acupuncture was effective in reducing anxiety, stress and fatigue levels, improving sleep latency in CAPS professionals in the post-COVID-19 pandemic context. There were no differences in muscle tension threshold with intervention. Most professionals were satisfied and considered the intervention necessary. Moreover, the general health status was rated as better after the end of the treatment, and the occurrence of adverse reactions was not significant.

## Data Availability

https://doi.org/10.48331/scielodata.88KFPC
