# Supplementary material for: Effectiveness of auricular laser acupuncture on anxiety, stress, sleep quality, fatigue and muscle tension of Psychosocial Care Center professionals: a quasi-experimental pilot study
Source: Rev Bras Enferm. 2025 Jul 11;78(3):e20240534. doi: 10.1590/0034-7167-2024-0534 (PMC12252929; doi:10.1590/0034-7167-2024-0534)
Supplement: Translation

# Translation: Efetividade da laser auriculoacupuntura na ansiedade, estresse, qualidade do sono, fadiga e tensão muscular de profissionais do Centro de Atenção Psicossocial: estudo piloto quase-experimental

**DOI:** 10.1590/0034-7167-2024-0534pt

**Authors:** Lara Rocha Silva, Luana Vieira Toledo, Andreia Guerra Siman, Talita Prado Simão Miranda, Maria Clara Vidgal Santana, Yara Martins Rodrigues, Bruna de Oliveira Alves, Caroline de Castro Moura

**Affiliations:** I Universidade Federal de Viçosa. Viçosa, Minas Gerais, Brasil; II Universidade Federal de Minas Gerais. Belo Horizonte, Minas Gerais, Brasil

**Keywords:** Acupuntura Auricular, Terapia com Luz de Baixa Intensidade, Serviço de Saúde Mental, Ansiedade, Estresse Psicológico.

## Abstract

**Objetivos:**

avaliar a efetividade da laser auriculoacupuntura em profissionais de Centros de Atenção Psicossocial quanto à ansiedade, estresse, qualidade do sono, fadiga, limiar de tensão do músculo trapézio, satisfação com o tratamento e possíveis reações adversas.

**Métodos:**

estudo piloto quase-experimental, realizado com 24 profissionais. Realizaram-se cinco sessões de laser ariculoacupuntura. Os dados foram coletados no início e no final do tratamento, e em um período de 15 dias após o encerramento (*follow-up*), analisados pelo teste de Wilcoxon emparelhado.

**Resultados:**

a laser auriculoacupuntura foi efetiva na redução dos níveis do estado de ansiedade, estresse, fadiga e melhora na latência do sono. Não houve diferença estatisticamente significativa no limiar de tensão muscular. Os profissionais ficaram satisfeitos com o tratamento, e a ocorrência de reações adversas não foi expressiva.

**Conclusões:**

a laser auriculoacupuntura desencadeou efeitos positivos para todas as variáveis investigadas, exceto limiar de tensão do músculo trapézio.

## INTRODUÇÃO

A pandemia de COVID-19 provocou mudanças significativas na sociedade, inclusive nos profissionais de saúde, que, devido à sobrecarga de trabalho e demandas físicas, intelectuais e emocionais, tiveram impacto negativo na sua qualidade de vida. Destacam-se, neste contexto, os profissionais do Centro de Atenção Psicossocial (CAPS), que, antes mesmo da pandemia, já apresentavam altos níveis de desordens emocionais, a exemplo da ansiedade^(1)^ e estresse^(2)^.

A ansiedade e estresse afetam a homeostasia corporal e ocasionam sinais e sintomas que são percebidos de formas diferentes pelos indivíduos. Entre eles, podem-se citar as alterações na qualidade do sono, fadiga e tensão muscular^(3)^.

Evidências científicas apontam alta prevalência de ansiedade e de estresse^(4)^ em profissionais de saúde durante a pandemia de COVID-19. Contudo, estudos que investigaram o impacto dessas desordens em profissionais dos CAPS durante o período pandêmico e pós-pandêmico são escassos. Nesse contexto, torna-se imprescindível conhecer o impacto da COVID-19 na saúde mental desses profissionais e como isso repercutiu no período pós-pandêmico, a fim de implementar medidas para a proteção e melhora da qualidade de suas vidas^(5,6)^. As Práticas Integrativas e Complementares em Saúde (PICS), que são institucionalizadas no Sistema Único de Saúde do Brasil^(7)^, em especial a auriculoacupuntura, têm se destacado neste cenário. A auriculoacupuntura é uma técnica terapêutica da acupuntura que consiste na estimulação de pontos energéticos presentes no pavilhão auricular, onde está representado todo o organismo, por meio de um microssistema^(8)^. Ao se estimular um ponto auricular, ocorre a liberação de peptídeos e neurotransmissores em via central, que desencadeiam a ação terapêutica da intervenção a nível sistêmico^(9)^. Tal técnica contribui para melhora da ansiedade, estresse, depressão^(10)^ e qualidade do sono^(11).^ Consequentemente, também auxilia na promoção da saúde mental.

Vários dispositivos podem ser utilizados para a estimulação dos acupontos auriculares, como cristais, agulhas, sementes e laser de baixa intensidade. O laser é um dispositivo não invasivo, asséptico e confortável para o usuário^(12)^. Contudo, a literatura científica é escassa sobre a utilização e a efetividade do laser auriculoacupuntura nas desordens emocionais em profissionais dos CAPS, apontando uma realidade científica pouco explorada.

## OBJETIVOS

Avaliar a efetividade da laser auriculoacupuntura em profissionais de CAPS quanto à ansiedade, estresse, qualidade do sono, fadiga, limiar de tensão do músculo trapézio, satisfação com o tratamento e possíveis reações adversas.

## MÉTODOS

### Aspectos éticos

O estudo foi conduzido de acordo com as diretrizes de ética nacionais^(13)^ e internacionais^(14)^, sendo aprovado pelo Comitê de Ética em Pesquisa da Universidade Federal de Viçosa, cujo parecer está anexado à presente submissão. O Termo de Consentimento Livre e Esclarecido foi obtido de todos os indivíduos envolvidos no estudo por meio escrito em duas vias, ficando uma com o pesquisador e a outra com o participante.

### Desenho, local do estudo e período

Estudo piloto quase-experimental, com avaliação de um grupo único antes e depois da intervenção, repostado segundo *Transparent Reporting of Evaluations with Nonrandomized Designs* (TREND) *statement* ^(15)^, e realizado com os profissionais dos CAPS I, II e AD III de duas cidades da Zona da Mata Mineira entre agosto de 2023 e julho de 2024.

### População e amostra; critérios de inclusão e exclusão

A população do estudo foi composta por todos os 53 profissionais que atuavam nos CAPS. A amostra foi obtida por conveniência, à medida que todos os profissionais foram convidados a colaborar com a pesquisa.

Foram incluídos profissionais com disponibilidade de horário para participar da intervenção e da coleta de dados. Foram excluídos aqueles profissionais que tinham perfurações na orelha, exceto no lóbulo auricular, histórico de fotossensibilidade, lesão, inflamação, tatuagem ou deformidade na orelha, uso de aparelhos auditivos, sistema imunológico comprometido, epilepsia, uso de marcapasso cardíaco, gestantes, puérperas ou mulheres que planejaram gestação durante o período de estudo, realização de outra PICS concomitante à realização da intervenção e licença ou férias durante a coleta de dados. Foram descontinuados do estudo aqueles profissionais que desenvolveram reação alérgica ou inflamatória no pavilhão auricular, perderam duas seções consecutivas ou excederem o intervalo maior que dez dias entre elas ou que não preencheram corretamente os instrumentos de coleta de dados.

### Protocolo do estudo

O recrutamento dos participantes ocorreu por meio de convite virtual e durante as reuniões de equipe dos profissionais. Aqueles que atenderam aos critérios de elegibilidade e aceitaram participar da pesquisa assinaram o Termo de Consentimento Livre e Esclarecido.

A auriculoacupuntura foi realizada por meio do laser de baixa intensidade de diodo, com comprimento de onda de 808 nanômetros (infravermelho), potência média de 100 mW e área do ponto do feixe de 1 cm^2^ (Therapy ACP - DCM^®^). O laser foi aplicado em contato com a pele, de forma contínua, pontual (perpendicular à pele) e estacionário. O tempo de aplicação foi de 40 segundos para cada ponto estimulado, sendo tempo total de aplicação da intervenção de seis minutos, com a dose energética de 4 joules (irradiância por ponto: 100 mW/cm^2^; fluência por ponto: 4,0 J/cm^2^)^(16)^. Um espaçador (área: 0,09842 cm^2^; diâmetro: 3,54 mm - DMC^®^) foi utilizado na ponta da cânula do laser com o objetivo de manter uma distância de segurança entre a cânula e a pele do participante. Antes da aplicação do laser, foi colocado filme de policloreto de vinila no espaçador e na cânula do equipamento para maior higiene, que era trocado a cada sessão. Durante toda a aplicação do laser, participante e intervencionista utilizaram óculos de proteção feitos de policarbonato com disposição de filme dielétrico especial para atenuação dos lasers.

Foram realizadas cinco sessões de laser auriculoacupuntura, uma por semana, com alternância de orelha em cada sessão. O participante foi posicionado sentado confortavelmente em uma cadeira, e foi realizada inspeção do pavilhão auricular a fim de detectar quaisquer alterações que impediam a aplicação da técnica. Em seguida, realizou-se antissepsia do local com álcool 70% e algodão. Um total de nove acupontos, validados cientificamente em profissionais de saúde com distúrbios emocionais no contexto pandêmico^(17)^, foram irradiados em todos os participantes, em todas as sessões: *Shenmen*; Sistema Nervoso Simpático; Rim; Fígado; Baço; Tronco Cerebral; Pulmão superior; Coração; e *Yang* do Fígado 2. Utilizou-se o mapa auricular preconizado pela *World Federation of Acupuncture-Moxibustion Societies* ^(18)^ para a correta localização dos acupontos. A intervenção foi realizada por enfermeiro especialista em auriculoacupuntura chinesa, com formação de seis meses na área, que foi devidamente treinado para a correta localização e irradiação dos acupontos por enfermeiro especialista em acupuntura com 12 anos de experiência na área.

A ansiedade e o estresse foram os desfechos primários deste estudo, e o sono, a fadiga e o limiar de tensão do músculo trapézio foram considerados desfechos secundários.

A ansiedade foi avaliada por meio do Inventário de Ansiedade Traço-Estado, que foi validado para o Brasil^(19)^ e avalia o estado emocional momentâneo. Conta com 20 itens com uma escala de Likert de quatro pontos, que varia de um (absolutamente não) a quatro (muitíssimo)^(19)^. O escore varia de 20 a 80 pontos, sendo baixo grau de ansiedade (20-30), grau mediano de ansiedade (31-49) e grau elevado de ansiedade (≥50)^(20)^.

O estresse foi mensurado por meio da *Perceived Stress Scale* ^(21)^, que foi traduzida e validada para o Brasil^(22)^. Nessa escala, são avaliados 14 itens, que variam de um (quase nunca) a quatro (sempre). O total do escore é dado pela soma das questões, podendo variar de zero a 56, sendo baixo nível de estresse (≤18), normal (19-24), moderado (25-29), alto (30-35) e muito alto (>35)^(22)^.

A qualidade do sono foi avaliada por meio do Índice de Qualidade de Sono de Pittsburgh, que foi traduzido e validado para o Brasil^(23)^. O questionário conta com 19 perguntas autoavaliadas, sendo cinco para serem respondidas por companheiro de cama ou quarto. As 19 questões são categorizadas nos componentes do sono, tais como: qualidade subjetiva, latência do sono, duração do sono, distúrbios do sono, uso de medicamentos e disfunção diária, e cada um é graduado em uma escala de zero a três. A soma do escore global varia de zero a 21, e pontuações acima de cinco indicam má qualidade do sono^(23)^. Para a presente pesquisa, utilizaram-se as 19 perguntas autoavaliadas.

A fadiga foi avaliada por meio da Escala de Avaliação da Fadiga, que foi traduzida para o português do Brasil^(24)^ e validada para o contexto de profissionais de saúde^(25)^. A escala é composta por dez itens com pontuações que variam de um (nunca) a cinco (sempre). A pontuação final é dada por meio da soma dos itens, podendo a variar de dez a 50. Quanto maior a pontuação, maiores são os sinais de fadiga^(24)^.

O limiar de tensão do músculo trapézio foi mensurado utilizando o algômetro digital (DDK, Kratos^®^)^(26)^. Para essa avaliação, o participante sentou em uma cadeira com a região peitoral apoiada no encosto da mesma, braços flexionados sobre o encosto, cabeça ereta, pernas descruzadas e pés totalmente apoiados no chão. Foi realizada pressão em quatro pontos da região do trapézio, de forma perpendicular à pele, nos seguintes locais: ponto médio do trapézio descendente (entre o acrômio e a 7ª vertebra cervical), bilateralmente; e ponto entre as 5ª e 6ª vértebras cervicais, no músculo trapézio descendente, bilateralmente. O participante foi solicitado a pressionar o cabo de interrupção do aparelho quando sentiu que o estímulo mecânico se tornou doloroso. Cada ponto foi avaliado em triplicata, e a média dos pontos foi utilizada para análise.

Foram consideradas, ainda, covariáveis do presente estudo: as variáveis de caracterização profissional, adaptadas de Assis *et al*.^(17)^; a influência da pandemia de COVID-19 na saúde mental e no rendimento profissional (não influenciou, influenciou para melhor ou influenciou para pior); o sentimento de sobrecarga laboral após a pandemia; a satisfação com o tratamento, por meio de três questões (satisfação com a intervenção, necessidade da intervenção e mudança no estado geral de saúde), em uma escala do tipo Likert de cinco pontos; e as possíveis reações adversas decorrentes da aplicação da intervenção (hiperemia, edema ou descamação na orelha e cefaleia), que foram avaliadas em uma escala de zero (nenhum desconforto) a dez (desconforto intenso).

A coleta de dados ocorreu em três momentos: antes de iniciar a intervenção (avaliação inicial); uma semana após a última sessão de intervenção (avaliação final); e após 15 dias da última intervenção (avaliação de *follow-up*). Os instrumentos foram autopreenchidos e as avaliações da tensão muscular foram realizados de forma presencial, pelo mesmo pesquisador, que foi devidamente treinado.

### Análise dos resultados e estatísticas

Os dados coletados foram analisados no *Statistical Package for the Social Sciences*, versão 23. Foi empregada a estatística descritiva para descrever e resumir as variáveis estudadas. Para verificar a distribuição dos dados, foi empregado o teste de Shapiro Wilk, o qual evidenciou a distribuição não normal das variáveis escalares. As variáveis de desfechos primários e secundários, em diferentes intervalos de tempo (avaliações inicial, final e *follow-up*), foram analisadas pelo teste de Wilcoxon emparelhado a 5% de significância. Para calcular o tamanho de efeito, foi utilizado o teste D de Cohen por meio do *software* GPower 3.1.9.2, que pode ser classificado como: *d*≤0,2 - tamanho de efeito pequeno ou nulo; 0,3<*d*<0,8 - tamanho de efeito médio; *d≥*0,8 - tamanho de efeito grade^(27)^.

## RESULTADOS

Dos 32 profissionais que atenderem aos critérios de elegibilidade, 24 (75,0%) concluíram o protocolo de intervenção até a etapa final (25,0% de perda), e 20 (62,5%), até a etapa de *follow-up* (37,5% de perda).

Dos 24 profissionais que completaram a intervenção, a maioria eram mulheres (66,7%), brancos (45,8%), solteiros (45,8%), católicos (70,8%) e não possuíam filhos (54,2%). A mediana da idade foi de 36 anos (20-62), e do tempo de serviço, 13,5 meses (6,2-52,5). Além disso, 45,8% trabalhavam no CAPS I, 29,2%, no CAPS II, e 25,0%, no CAPS AD III. Destes, 54,2% possuíam dois ou mais empregos, e 33,3% eram efetivos. Adicionalmente, 91,7% trabalharam durante a pandemia de COVID-19, e 54,2% contraíram a doença. Quanto às condições clínicas, 16,7% possuíam algum transtorno de ansiedade; 8,3% utilizavam ansiolítico; 20,8% utilizavam antidepressivos; 66,7% consumiam álcool; e 29,2% eram tabagistas. Além disso, 58,3% dos profissionais relataram que a pandemia influenciou negativamente a sua saúde mental; 29,2% relataram que a pandemia influenciou negativamente o rendimento profissional; e 41,7% se sentiram mais sobrecarregados no trabalho após a pandemia.

Verificou-se redução estatisticamente significativa no nível de ansiedade-estado entre as avaliações inicial e de *follow-up*, com tamanho de efeito médio. Houve também redução estatisticamente significativa no nível de estresse entre as avaliações inicial e final e inicial e de *follow-up,* apresentando tamanho de efeito médio em ambas as avaliações. Na avaliação da qualidade do sono, também foi evidenciada melhora estatisticamente significativa no componente latência do sono entre as avaliações inicial e final, com tamanho de efeito pequeno. Ainda, ocorreu melhora estatisticamente significativa da fadiga entre o início e o final do tratamento, com tamanho de efeito médio. Não ocorreram mudanças estatisticamente significativas na tensão do músculo trapézio ao longo do tratamento (Tabela 1).

**Tabela 1:** Níveis de ansiedade-estado, estresse, fadiga, sono e tensão muscular nas avaliações inicial, final e *follow-up* (n=24), Viçosa, Minas Gerais, Brasil, 2024

	AI n=24		AF n=24		AFW n=20		Valor de *p* ^||^ (tamanho de efeito)		
**Variável**	**Média** **(DP)**	**Mediana** **(DI)**	**Média** **(DP)**	**Mediana** **(DI)**	**Média** **(DP)**	**Mediana** **(DI)**	**AF** **AI**	**AFW** **AF**	**AFW** **AI**
**Ansiedade estado**	45,13 (9,65)	45,00 (38,75-50,50)	42,42 (9,93)	41,00 (36,00-51,00)	39,65 (9,36)	39,50 (33,25-46,75)	0,053 (0,272)	0,730 (0,286)	0,017 (0,566)
**Estresse**	30,71 (9,10)	30,50 (23,75-38,00)	25,67 (7,90)	27,00 (19,00-33,00)	24,35 (6,74)	25,00 (18,25-30,75)	0,001 (0,588)	0,635 (0,178)	0,005 (0,777)
**Qualidade do sono**									
Qualidade global do sono	8,54 (3,98)	9,00 (6,00-10,00)	7,36 (3,88)	7,00 (4,50-9,75)	7,45 (3,33)	8,00 (5,25-9,00)	0,560 (0,300)	0,750 (0,024)	0,216 (0,294)
Qualidade subjetiva do sono	1,46 (0,78)	1,00 (1,00-2,00)	1,33 (0,64)	1,00 (0,00-3,00)	1,20 (0,52)	1,00 (1,00-1,75)	0,405 (0,180)	0,414 (0,339)	0,317 (0,377)
Latência do sono	1,71 (1,20)	2,00 (0,25-3,00)	1,38 (1,01)	1,50 (0,25-2,00)	1,40 (0,99)	1,00 (1,00-2,00)	0,035 (0,295)	0,655 (0,019)	0,096 (0.279)
Duração do sono	1,04 (0,86)	1,00 (0,00-2,00)	0,88 (0,74)	1,00 (0,00-1,00)	0,95 (0,83)	1,00 (0,00-1,00)	0,285 (0,198)	0,180 (0,088)	0,729 (0,106)
Eficiência do sono	0,71 (0,75)	1,00 (0,00-1,00)	0,79 (0,93)	0,50 (0,00-1,75)	0,95 (1,05)	0,50 (0,00-2,00)	0,644 (0,093)	0,257 (0,160)	0,417 (0,256)
Distúrbio do sono	1,79 (0,93)	2,00 (1,00-2,00)	1,54 (0,78)	1,00 (1,00-2,00)	1,55 (0,60)	1,50 (1,00-2,00)	0,083 0,289	0,414 (0,014)	0,134 (0,293)
Uso de medicação	0,63 (1,13)	0,00 (0,00-1,00)	0,54 (0,98)	0,00 (0,00-1,00)	0,40 (0,94)	0,00 (0,00-0,00)	0,726 (0,084)	0,414 (0,145)	0,581 (0,219)
Sonolência e disfunção diurna	1,21 (0,93)	1,00 (0,25-2,00)	1,00 (0,78)	1,00 (0,25-1,00)	1,00 (0,72)	1,00 (0,25-1,75)	0,212 (0,242)	0,705 (0,001)	0,405 (0,248)
**Fadiga**	24,83 (5,35)	24,00 (20,25-28,75)	22,79 (4,36)	22,50 (20,00-25,00)	22,50 (3,65)	22,00 (21,00-24,00)	0,020 (0,413)	0,625 (0,123)	0,190 (0,492)
**Tensão no músculo trapézio**	4,17 (1,40)	4,14 (3,18-5,30)	3,80 (1,38)	3,84 (2,97-5,08)	3,82 (1,49)	3,86 (2,60-5,40)	0,103 (0,266)	0,884 (0,01)	0,108 (0,241)

AI - avaliação inicial; AF - avaliação final; AFW - avaliação de follow-up; n - frequência absoluta;

|| teste de Wilcoxon emparelhado; DP - desvio padrão; DI - distância interquartílica.

A maior parte dos profissionais (79,1%) ficou satisfeita ou extremamente satisfeita com a intervenção, e considera o tratamento necessário ou totalmente necessário (79,1%). Ainda, após o término, 62,5% consideram que o seu estado de geral de saúde estava melhor (Tabela 2).

**Tabela 2:** Satisfação, necessidade da intervenção e estado geral de saúde dos participantes do estudo (n=24), Viçosa, Minas Gerais, Brasil, 2024

Variáveis	n	%
Satisfação com a intervenção		
Extremamente insatisfeito	0	0,0
Insatisfeito	0	0,0
Não tenho certeza	5	20,8
Satisfeito	14	58,4
Extremamente satisfeito	5	20,8
Necessidade da intervenção		
Totalmente desnecessária	0	0,0
Desnecessária	0	0,0
Não tenho certeza	5	20,8
Necessária	14	58,4
Totalmente necessária	5	20,8
Estado geral de saúde após a intervenção		
Muito pior	0	0,0
Pior	0	0,0
Nenhuma mudança	9	37,5
Melhor	15	62,5
Muito melhor	0	0,0

*n - frequência absoluta; % - frequência relativa.*

Por fim, apenas um profissional (4,2%) relatou cefaleia (intensidade=2/10) após a segunda sessão de laser auriculoacupuntura. Os demais (95,8%) não apresentaram reações adversas.

## DISCUSSÃO

Evidenciou-se, com a realização do presente estudo, que a laser auriculoacupuntura foi capaz de reduzir os níveis de ansiedade-estado, estresse e fadiga, além de proporcionar melhora na qualidade do sono, especificamente no componente latência do sono, em profissionais dos CAPS, no contexto pós-pandemia de COVID-19, durante o período de tratamento estabelecido. Grande parte dos profissionais ficaram satisfeitos com a intervenção realizada, considerando-a necessária e relatando melhora do seu estado geral de saúde após o tratamento. Além disso, a ocorrência de reações adversas não foi expressiva.

Os achados do presente estudo são relevantes, pois envolveram um público que é pouco investigado na literatura científica, especialmente em relação ao acometimento de desordens emocionais. Além disso, a utilização do laser é uma inovação no tratamento por meio da auriculoacupuntura, e sua atuação em fatores emocionais é pouco explorada. De fato, revisão sistemática^(10)^ com objetivo de identificar os efeitos da auriculoacupuntura no tratamento do estresse, ansiedade e depressão revelou que, dos 24 artigos apresentados, apenas um abordou a laser auriculoacupuntura para sintomas físicos e emocionais da disfunção temporomandibular^(16)^, evidenciando que esta é uma área que ainda necessita de investigação.

A neurofisiologia explica a ação da auriculoacupuntura através da modulação do eixo hipotálamo-hipófise^(28)^. Acredita-se que a estimulação dos acupontos desencadeie sinais bioquímicos que promovam mudanças em estruturas cerebrais, como no sistema límbico e no córtex pré-frontal^(29)^, contribuindo para a redução da ansiedade e estresse, por exemplo.

O agravamento da ansiedade foi um dos efeitos deletérios na saúde mental dos profissionais de saúde durante a pandemia de COVID-19^(30)^, e seus efeitos ainda são perceptíveis. Após a laser auriculoacupuntura, houve redução estatisticamente significante de 12,1% nos níveis do estado de ansiedade entre os momentos iniciais e de *follow-up*. Os achados corroboram estudo clínico randomizado que utilizou o mesmo protocolo de pontos auriculares e número de sessões, desenvolvido com 284 profissionais de enfermagem durante a pandemia, os quais apresentaram redução de 60% na ansiedade entre os momentos iniciais e finais e 35,9% entre os momentos iniciais e de *follow-up*, utilizando agulhas^(17)^. Nesse sentido, o efeito desencadeado pelas agulhas, que são consideradas os dispositivos padrão-ouro na acupuntura, parece ser superior na redução da ansiedade; entretanto, são necessários estudos que comparem essas intervenções, na mesma população, para que se possa estabelecer essa relação.

O estresse apresentou redução estatisticamente significativa entre os momentos inicial e final e inicial e de *follow-up*, de 16,4% e 20,7%, respectivamente, passando do nível alto para moderado após o tratamento. Pesquisa com profissionais de enfermagem na pandemia revelou redução de 38,3% nesta variável em uma única sessão de auriculoacupuntura com sementes, utilizando-se os pontos *Shenmen*, Rim, Sistema Nervoso Simpático, Alegria, Ansiedade, Antidepressivo, Coração, Endócrino, Pulmão e Relaxamento Muscular^(31)^. Estudo de metodologia mista, com abordagem quantitativa randomizada e qualitativa exploratória com a equipe de enfermagem perioperatória, evidenciou redução no estresse de 34,0% após o tratamento e 19,9% 15 dias após a intervenção, em comparação ao momento inicial^(32)^. Tal estudou utilizou agulhas semipermanentes nos acupontos *Shenmen,* Tronco Cerebral, Rim, Simpático e Fígado, em oito sessões, duas vezes por semana^(32)^. Destaca-se que os acupontos *Shenmen*, Rim e Sistema Nervoso Simpático foram comuns nesses estudos. O *Shenmen* possui função sedativa; o Rim função energética e revigorante; e o Sistema Nervoso Simpático é responsável pela regulação dos sistemas nervosos simpático e parassimpático, com efeito sobre o relaxamento muscular e a dor^(10)^. Nesse sentido, a combinação dos três pontos, utilizando sementes, agulhas, ou laser, apresenta resultados positivos para o estresse.

Em relação à qualidade do sono, observou-se redução estatisticamente significativa de 18,8% do componente latência do sono entre os momentos inicial e final. Vale salientar que os dois acupontos utilizados no presente estudo, *Shenmen* e Coração, contribuem para melhora do sono, uma vez que estimulam o nervo vago e promovem a ação parassimpática no organismo^(33)^, contribuindo para relaxamento necessário para o sono. De fato, estudo quase experimental com enfermeiros hospitalares durante a pandemia demonstrou redução de 52,3% na qualidade global do sono e 37,5% na média da latência do sono, utilizando esferas neutras com três sessões nos pontos *Shenmen*, Rim, Sistema Nervoso Simpático, Coração, Pulmão, Subcórtex, Endócrino, Ponto Zero ou Relaxamento Muscular, e Adrenal^(11)^. Na China, estudo randomizado duplo-cego com 147 idosos identificou que a magneto auriculoacupuntura, a laser auriculoacupuntura e a combinação de ambas as terapias, nos acupontos *Shenmen,* Coração, Fígado, Baço, Rim, Occipital e Subcórtex, três vezes por semana, durante seis semanas, reduziu significativamente o Indice de Qualidade de Sono de Pittsburg, sendo significativo entre o momento inicial e o sexto mês de *follow-up* na população estudada^(34)^. Neste estudo^(34)^, utilizou-se o laser de baixa densidade, de modo contínuo, com comprimento de onda de 650 nm, potência média de saída de 2,5 mW, densidade de energia de 0,54 J/cm^2^ por um minuto, e cada ponto foi irradiado por cerca de 60 segundos. Apesar de o estudo apresentar parâmetros de laser diferenciados da presente pesquisa, cabe observar que ambos exerceram efeitos positivos sobre a qualidade do sono. Ademais, parece que a terapêutica com mais sessões e menor intervalo entre elas é mais benéfica para melhorar esta variável e perdurar os seus efeitos. Entretanto, para tal afirmação, sugere-se a realização de estudos clínicos que comparem diferentes parâmetros dosimétricos do laser com o mesmo protocolo auricular.

A má qualidade do sono pode desencadear fadiga^(35)^, e esta pode afetar o bem-estar psicológico dos profissionais, como aconteceu durante a pandemia de COVID-19^(36)^. De fato, estudo multicêntrico descritivo e transversal, desenvolvido com 2.667 enfermeiros durante a pandemia, identificou relação direta entre o grau de ansiedade, estresse percebido, depressão e fadiga^(37)^. Com a presente pesquisa, evidenciou-se redução estatisticamente significativa de 8,2% na fadiga ao final do tratamento. Tais achados, nesse contexto, podem ser justificados pela melhora dos níveis de estresse e ansiedade, por meio da laser auriculoacupuntura, que pode ter contribuído para melhorar a disposição dos profissionais. Destaca-se que, até o presente momento, não foi identificado na literatura científica estudo utilizando tal técnica sobre os níveis de fadiga em profissionais de saúde, evidenciando o caráter inovador deste estudo. Cabe salientar a importância de adotar medidas para controlar a fadiga no ambiente de trabalho e, consequentemente, doenças ocupacionais.

O aumento da tensão muscular é uma das manifestações corporais ocasionadas pelas desordens emocionais. A auriculoacupuntura mostrou melhora significativa nos padrões do algômetro em dores nas costas com protocolo de cinco sessões, uma vez por semana, com a utilização de agulhas^(38)^. Os acupontos utilizados foram *Shenmen*, Rim, Sistema Nervoso Simpático, Subcórtex, Bexiga, Fígado e aqueles correspondentes ao local da dor^(38)^. Cabe salientar que, em tal investigação^(38)^, os acupontos selecionados possuem ação analgésica e de relaxamento muscular, o que pode ter ocasionado melhor efeito terapêutico sobre o limiar de dor. Nesse sentido, sugere-se que futuras pesquisa incluam acupontos com essas funções, a fim de verificar a ação da laser auriculoacupuntura no limiar de tensão muscular.

Ressalta-se, ainda, que a maioria dos profissionais relataram ficar satisfeitos com a intervenção realizada, que é uma terapêutica necessária e que produz melhora no estado geral de saúde. Em consonância, estudo realizado com estudantes durante a pandemia também demonstrou grande aceitabilidade da técnica^(39)^, permitindo a sua implementação nessa população. Estudo qualitativo com profissionais de enfermagem demonstrou que o fato de o procedimento ocorrer no ambiente de trabalho facilitou a realização da intervenção e que esses se sentiram reconhecidos e valorizados^(40)^. Este fato, somado à utilização do laser, o qual permite que a terapêutica seja indolor, não invasiva^(41)^ e confortável^(12)^, pode ter contribuído para esses achados. A alta satisfação com o tratamento realizado, associado aos efeitos desencadeados pela intervenção, pode se configurar como importantes indicadores para favorecer a sua implementação nos ambientes laborais como parte de políticas públicas em prol da saúde do trabalhador.

Além disso, nenhum participante apresentou evento adverso grave. Apenas um participante apresentou cefaleia, a qual foi transitória, tolerável e de baixa intensidade, possibilitando a continuidade do tratamento. Estudo randomizado, com o objetivo de verificar se a magneto e a laser auriculoacupuntura eram efetivas para a melhora do sono, identificou como eventos adversos da laser ariculoacupuntura o prurido no ouvido e sensibilidade dos acupontos, os quais foram resolvidos espontaneamente^(34)^. Nesse sentido, o uso do laser na auriculoacupuntura vem se consolidando como uma prática segura, sendo associada a poucos eventos adversos, leves e transitórios^(42)^. Além do mais, é inquestionável a sua efetividade^(12)^.

Os achados desta pesquisa reforçam que a auriculoacupuntura é benéfica na redução da ansiedade e estresse em profissionais de saúde. Destacam-se como pontos positivos a dosagem precisa do laser, a exposição de uma pequena área, a facilidade no seu uso, o tempo mínimo necessário para a realização do procedimento e a facilidade no seu uso^(12)^. Além disso, é asséptica, confortável, não invasiva^(12)^ e indolor^(42)^. Essa combinação de benefícios torna a laser auriculoacupuntura uma terapêutica promissora para o cuidado da saúde mental dos profissionais de saúde, com possibilidade de ser implementada em programas de promoção da saúde do trabalhador.

### Limitações do estudo

Como limitação do presente estudo, evidenciam-se o tamanho da amostra e o seu recrutamento por conveniência, o que pode interferir na generalização dos achados. Contudo, este foi estudo piloto conduzido com uma população ainda pouco investigada, que pode fornecer subsídios para futuros ensaios clínicos, com amostras mais expressivas. Além disso, a alta taxa de desistência dos profissionais, principalmente por desligamentos e atestados, restringiu o acompanhamento de um número maior de participantes. Outra limitação refere-se à sobrecarga dos profissionais, decorrente da composição incompleta das equipes, o que impediu a realização de entrevistas durante a coleta de dados. Para contornar essa situação, adotou-se o autopreenchimento, contudo algumas perdas amostrais ocorreram nesse momento.

### Contribuição para as áreas da saúde, enfermagem ou políticas públicas

Os achados do estudo fornecem subsídios para que o enfermeiro possa incorporar a laser auriculoacupuntura no seu contexto de cuidado. A apropriação de tal prática milenar permite o olhar holístico sobre a pessoa. Além disso, a utilização do laser oferece uma terapêutica segura, indolor e de alta aceitação, viabilizando o tratamento em massa, pois requer poucos materiais para a sua implementação. No contexto de trabalho do CAPS, essa abordagem melhora as desordens emocionais, contribuindo para bem-estar emocional dos profissionais ao lidar com situações que podem comprometer a sua saúde mental.

## CONCLUSÕES

A laser auriculoacupuntura foi efetiva na redução dos níveis do estado de ansiedade, estresse e fadiga, melhorando o componente latência do sono em profissionais do CAPS no contexto pós-pandemia de COVID-19. Não houve diferenças no limiar de tensão muscular com a intervenção. A maior parte dos profissionais ficou satisfeita e considerou a intervenção necessária. Além disso, o estado geral de saúde foi classificado como melhor após o término do tratamento, e a ocorrência de reações adversas não foi expressiva.

## Data Availability

https://doi.org/10.48331/scielodata.88KFPC
